# Deoxysphingoid bases as plasma markers in Diabetes mellitus

**DOI:** 10.1186/1476-511X-9-84

**Published:** 2010-08-16

**Authors:** Mariana Bertea, Markus F Rütti, Alaa Othman, Jaqueline Marti-Jaun, Martin Hersberger, Arnold von Eckardstein, Thorsten Hornemann

**Affiliations:** 1 Institute for Clinical Chemistry, University Hospital Zurich and Center for Integrative Human Physiology, University of Zurich, Zurich, Switzerland; 2 Competence Center for Systems Physiology and Metabolic Diseases, Zurich, Switzerland; 3 Divison of Internal Medicine, Hospital Wil, Wil, Switzerland; 4 Division of Clinical Chemistry and Biochemistry, University Children's Hospital Zurich and Center for Integrative Human Physiology, University of Zurich, Zurich, Switzerland

## Abstract

**Background:**

Sphingoid bases are formed from the precursors L-serine and palmitoyl-CoA-a reaction which is catalyzed by the serine-palmitoyltransferase (SPT). SPT metabolizes, besides palmitoyl-CoA also other acyl-CoAs but shows also variability towards the use of other amino acid substrates. The enzyme is also able to metabolize alanine, which results in the formation of an atypical deoxy-sphingoid base (DSB). This promiscuous activity is greatly increased in the case of the sensory neuropathy HSAN1, and pathologically elevated DSB levels have been identified as the cause of this disease. Clinically, HSAN1 shows a pronounced similarity to the diabetic sensory neuropathy (DSN), which is the most common chronic complication of diabetes mellitus. Since serine and alanine metabolism is functionally linked to carbohydrate metabolism by their precursors 3-phosphoglycerate and pyruvate, we were interested to see whether the levels of certain sphingoid base metabolites are altered in patients with diabetes.

**Results:**

In a case-control study we compared plasma sphingoid base levels between healthy and diabetic individuals. DSB levels were higher in the diabetic group whereas C16 and C18 sphingoid bases were not significantly different. Plasma serine, but not alanine levels were lower in the diabetic group. A subsequent lipoprotein fractionation showed that the DSBs are primarily present in the LDL and VLDL fraction.

**Conclusion:**

Our results suggest that DSBs are a novel category of plasma biomarkers in diabetes which reflect functional impairments of carbohydrate metabolism. Furthermore, elevated DSB levels as we see them in diabetic patients might also contribute to the progression of the diabetic sensory neuropathy, the most frequent complication of diabetes.

## Introduction

**Figure 1:**
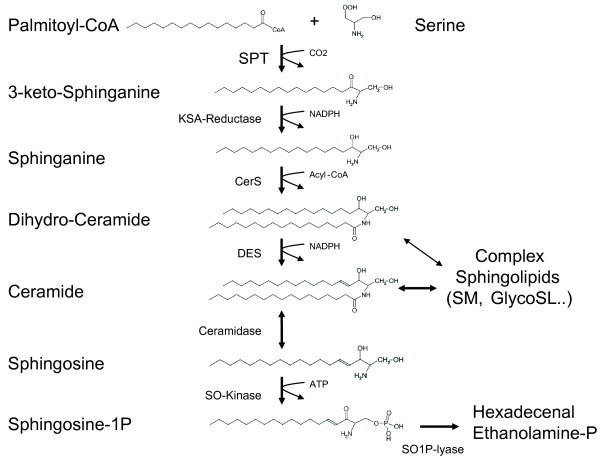
**De-novo sphingolipid synthesis pathway**. De-novo ceramide synthesis involves several steps. Serine Palmitoyltransferase (SPT) catalyzes the initial conjugation of palmitoyl-CoA with L-serine to form 3-keto-sphinganine which is subsequently reduced to sphinganine (SA). SA is acetylated by ceramide synthase (CerS) and desaturated by ceramide desaturase (DES) to form ceramide. The degradation pathway starts with the deacetylation of ceramide by ceramidase. The sphingosine (SO) formed is then phosphorylated by SO-Kinase and finally degraded to hexadecenal and phosphoethanolamine by the action of the sphingosine-1-phospate lyase (SO1P-lyase).

Sphingolipids comprise a heterogeneous class of lipids that contribute to plasma membrane and plasma lipoprotein formation. They are derived from the aliphatic amino-alcohol sphingosine, which is commonly formed from the precursors L-serine and palmitoyl-CoA. The condensation of serine with palmitoyl-CoA is a pyridoxalphosphate (PLP) dependent reaction and catalyzed by the enzyme serine palmitoyltransferase (SPT) (EC 2.3.1.50). SPT is a heteromeric enzyme and composed of at least three subunits (SPTLC1, SPTLC2 and SPTLC3) [1,2]. The SPTLC2 and SPTLC3 subunits comprise a PLP consensus sequence which is absent in the SPTLC1 subunit. The product of the SPT reaction, 3-keto-sphinganine, is converted to sphinganine (SA) and subsequently N-conjugated with a second fatty acid to form dihydro-ceramide (figure 1). The majority of dihydro-ceramide is then desaturated at C4 to form ceramide, which is the building block for the more complex sphingolipids. Ceramide and to a certain extent also dihydro-ceramide is usually O-linked to a polar head group such as phosphocholine or carbohydrates. This leads to a complex variety of different sphingolipid metabolites. Although L-serine and palmitoyl-CoA are the preferred substrates, the enzyme shows a certain flexibility towards the use of other substrates. Besides palmitoyl-CoA, SPT also metabolizes other acyl-CoAs with a carbon chain length of between C_12 _and C_18_. In this, the SPTLC3 subunit shows a higher affinity towards shorter acyl-CoAs (e.g. C_12 _and C_14_) whereas SPTLC2 shows a higher activity with C_16 _and C_18 _acyl-CoAs. Both C_18 _sphingoid and C_16 _sphingoid bases have been detected in significant amounts in human plasma [3].

Moreover, SPT shows variability towards the use of other amino acid substrates. Besides L-serine, which is the preferred substrate, the enzyme also metabolizes L-alanine and to a certain extent glycine [4,5]. This generates an atypical category of sphingoid bases: the 1-deoxy-sphingoid bases (DSBs). The conjugation of alanine forms deoxy-sphinganine (doxSA), whereas the use of glycine results in the formation of deoxymethyl-sphinganine (doxmethSA). Both metabolites are devoid of the C_1_-hydroxyl group of SA and are therefore neither metabolized to complex sphingolipids nor degraded by the regular sphingolipid catabolism, since sphingosine-1P as a catabolic intermediate cannot be formed from DSBs.

The activity of SPT with alanine and glycine is greatly increased in the presence of several SPT missense mutations which are associated with the inherited sensory neuropathy HSAN1 (OMIM162400). HSAN1 is an autosomal dominantly inherited axonal neuropathy that is clinically characterized by a loss of pain and temperature sensation, usually starting in the lower limbs and often accompanied by neuropathic pain attacks and skin ulcers. The mutant SPT in HSAN1 shows a highly increased activity with alanine and glycine compared to the wildtype SPT. Consequently, these lipids are found at elevated levels in cells and plasma from HSAN1 patients [4]. Significantly elevated DSB levels were also found in plasma and PNS tissue of the HSAN1 mouse model [6]. HSAN1 mice are transgenic for the mutant SPT and develop a sensory neuropathy within 6-9 months of age. In contrast, double transgenic mice which concomitantly co-express mutant and wildtype SPT are protected and show, in parallel, also significantly lower tissue and plasma DSB levels [6]. The addition of doxSA to DRGs resulted in a dose-dependent reduction of neurite formation *in-vitro *and in a disruption of the neuronal cytoskeleton structure [4].

Originally, doxSA (also referred to as "Spisulosine" or "ES-285") has been isolated from the arctic clam *Spisula polynyma *as an investigational marine anticancer drug. It was shown to induce cell death in various breast cancer cell lines [7,8] and to interfere with stress fibre formation and cytoskeleton dynamics by affecting Rho/Rac-GTPase signaling cascades [9].

Clinically, HSAN1 shows a pronounced similarity to the diabetic sensory neuropathy (DSN), which is the most common chronic complication of diabetes mellitus. Both diseases have a late onset and slow progression and typically affect the distal extremities first. The degeneration of small sensory fibers results in the loss of pain sensation, which in turn leads to painless injuries [10].

In this context it is important to note that DSBs are not exclusively formed by the mutant but also by the wildtype SPT - however to a lesser extent [11]. Comparing the kinetics between mutant and wildtype SPT showed that both forms have similar Km's for serine and alanine but that Vmax for alanine is greatly increased in the HSAN1 mutants [12]. Significant DSB levels are therefore also detected in the plasma of healthy individuals [4]. Interestingly, serine and alanine metabolism is physiologically linked to the cellular carbohydrate metabolism. Serine is formed from 3-phosphoglycerate and alanine from pyruvate, which are both intermediates of the glycolytic chain. Within this framework of evidence, we were interested to investigate whether sphingoid base levels are altered in patients with an impaired glucose metabolism such as that seen in diabetes mellitus.

## Materials and methods

### Patients

A total of 70 men and 29 women from Zurich volunteered to participate in the study. Written informed consent was obtained from all participants and the local Ethics Committees approved the study [13]. The case group consisted of 50 consecutive Caucasian patients with diagnosed diabetes mellitus type II. The control group consisted of 49 Caucasians with no history of diabetes recruited from the general population. Clinical chemistry analysis was carried out on a Roche-Hitachi Modular Clinical Chemistry analyzer using commercial tests from Roche Diagnostics (Rotkreuz, Switzerland).

### Lipid extraction, hydrolysis and LC-MS analytic

Plasma sphingolipids were extracted and analyzed by LC-MS as described earlier [4]

### Separation of plasma lipoproteins

Plasma was isolated from diabetic donors after overnight fasting. Three ml plasma was fractionated on a four step density gradient essentially as described [14]. Ultracentrifugation was performed in a Beckman SW-40 swinging bucket rotor for 24 h at 41000 rpm at 15°C. Fractions (1 ml) were collected from the top of the centrifuge tube and analyzed for triglyceride; cholesterol and sphingoid bases were analyzed as described above.

### Amino acid analysis

Plasma amino acid concentrations were analyzed according to the method of Frank and Powers [15]

### Statisics

Significance levels were determined with an unpaired student t-test using Sigma Plot v10.0 (SystatSoftware, Inc.). The correlation matrix was generated in SPSS (SPSS, Switzerland). ROC analysis was performed with XLSTAT (Addinsoft, Inc.)

## Results

Sphingolipids in plasma are usually present in a broad variation of subspecies. Sphingoid bases can be found in the saturated (sphinganine, dihydro-sphingosine) and unsaturated (sphingosine) state and are usually N-acetylated with a second fatty acid. Most of the plasma sphingolipids are also conjugated to O-linked head groups, which divert them analytically into various subgroups. However, for this study we were primarily interested in differences between the individual sphingoid base backbones. We therefore simplified the analysis by subjecting the sphingolipids to a sequential acid and base hydrolysis. Acid hydrolysis specifically breaks the N-alkyl chain, whereas alkaline conditions lead to a release of the O-linked head group. The resulting free sphingoid base metabolites were analyzed by LC-MS. The sum of the individual sphingoid bases reflects the total sphingolipid content in the analyzed samples.

In this study we analyzed the C_16_-, C_18_-and deoxy-sphingolipid levels in a case-control setup comparing 50 patients with documented diabetes mellitus type II (D) and 49 healthy controls (C). The average age of the control group was slightly younger and included more females than the diabetic group. Results are summarised in table 1.

**Table 1:** Baseline characteristics and sphingoid base concentrations for the control and diabetic group

	Control	Diabetes	P-value
	**N = 49**	**N = 50**	

			

AGE(y)	57.84 ± 11.64	63.31 ± 7.43	0.01

**BMI**	**24.83 ± 4.00**	**28.97 ± 4.5**	**< 0.0001**

Male (%)	54	84	

**CHOL (mM)**	**5.79 ± 1.26**	**4.82 ± 1.11**	**< 0.0001**

**HDL (mM)**	**1.64 ± 0.5**	**1.30 ± 0.33**	**< 0.0001**

**LDL (mM)**	**3.47 ± 0.97**	**2.90 ± 0.95**	**0.004**

TG (mM)	1.48 ± 0.93	1.44 ± 0.67	0.77

CRP (mg/l)	2.01 ± 2.18	2.72 ± 4.14	0.28

SMOKING (%)	42	77	

STATINS (%)	2	70	

			

C16SO (μM)	11.06 ± 5.40	10.84 ± 8.71	0.88

C16SA (μM)	0.35 ± 0.18	0.30 ± 0.21	0.20

C18SO (μM)	79.44 ± 28.58	72.93 ± 38.76	0.34

C18SA (μM)	2.52 ± 0.91	2.22 ± 1.14	0.14

**doxSO (μM)**	**0.12 ± 0.09**	**0.19 ± 0.15**	**0.005**

**doxSA (μM)**	**0.06 ± 0.04**	**0.08 ± 0.05**	**0.08**

As reported earlier, the most abundant sphingoid bases in plasma were C_18_-Sphingosine (C_18_SO) and C_16_-Sphingosine (C_16_SO). The concentrations of the dihydro-forms C_18_-Sphinganine (C_18_SA) and C_16_-Sphinganine (C_16_SA) were much lower and represented about 1-5% of the respective sphingosine forms. Deoxy-sphingoid bases were generally minor, representing 0.1-0.5% of the total plasma sphingoid bases. For the DSBs the saturated (doxSA) and unsaturated (doxSO) metabolites were found at similar concentrations. Deoxymethyl-SA or deoxymethyl-SO were not detected.

**Figure 2:**
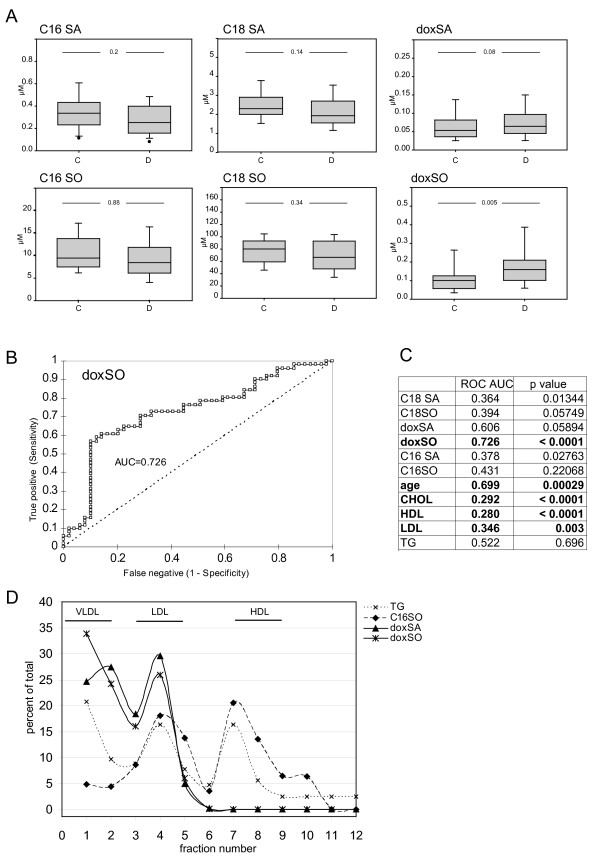
**A) Boxplot representation of C_16_, C_18 _and DSB levels in the control (C) and diabetic (D) cohort**. Whereas C_16_SA, C_16_SO, C_18_SA and C_18_SO were not different between the two groups were doxSA and doxSO levels higher in the diabetic group. (Box represents the upper and lower quartile, whiskers show the 5% and 95% percentile, the horizontal line represents the median) C) Receiver operator curve (ROC) for doxSO D) Area under the curve (AUC) and p-values for all analyzed variables were calculated from a ROC analysis. Most significant parameters are marked (p values were calculated with the null hypothesis (H0) as AUC = 0.5). E) Distribution of the DSB and C16 sphingoid bases in the distinct lipoprotein fractions. Human plasma was separated by a four step density gradient ultracentrifugation. The individual fractions were assayed for cholesterol (not shown), triglycerides (TG; dashed line), doxSA (triangle) doxSO (triangle) and C_16_SO. Both doxSA and doxSO are only found in the VLDL and LDL but not in the HDL lipoprotein fraction. C_16_-SO, in contrast, was present in LDL and HDL but not in VLDL. The sum of all fractions was defined as 100% and values are given as a percentage of the total.

We found that the average doxSO levels were significantly higher in the diabetic than in the control group. Also doxSA levels were, although less pronounced, higher in the diabetic group (table 1, figure 2A). On the other hand, C_16 _and C_18 _sphingoid base levels were not significantly different between the two groups. This indicates that certain sphingoid bases are specifically altered under diabetic conditions whereas others remain unchanged. Among the other variables total-and LDL-cholesterol was lower in the diabetic groups, which is explained by the general administration of cholesterol lowering drugs (e.g. statins) in this group. HDL cholesterol was lower in the diabetic groups whereas triglycerides were not different. Age and smoking had no influence.

Since doxSO showed the most significant changes between the groups we calculated the diagnostic values for this variable. A receiver operator curve (ROC) analysis (figure 2B) revealed an area under the curve (AUC) of 0.726. This converts into a sensitivity of 61% and a specificity of 89% at a doxSO threshold of 0.2 μM. The AUC for the other variables is summarized in figure 2C.

C_16 _and C_18_-sphingoid bases are functionally linked to serine metabolism whereas the DSBs are linked to alanine metabolism. We therefore also analyzed the plasma amino acid levels between the two groups. Total amino acid concentration was in general lower in diabetic patients. The most significant changes were seen for glycine, serine and threonine, whereas other amino acids like alanine, valine or histidine were not altered (see table 2).

**Table 2:** Average plasma amino acid concentrations in the control and diabetes group.

	Control	Diabetes	P-value
	**N = 49**	**N = 50**	

			

**Glycine**	**412.88 ± 162.50**	**305.42 ± 92.80**	**0.0001**

Alanine	408.91 ± 146.71	365.24 ± 126.73	0.12

**Serine**	**151.83 ± 52.84**	**122.73 ± 34.64**	**0.001**

Valine	646.17 ± 260.35	587.32 ± 186.66	0.20

**Threonine**	**119.70 ± 44.43**	**86.99 ± 26.53**	**< 0.0001**

**Isoleucine**	**85.45 ± 37.29**	**69.30 ± 22.56**	**0.009**

**Leucine**	**153.52 ± 58.83**	**131.04 ± 44.77**	**0.03**

**Asparagine**	**119.13 ± 58.30**	**88.43 ± 33.58**	**0.002**

Aspartate	25.84 ± 12.18	23.38 ± 11.56	0.30

**Glutamine**	**339.60 ± 231.49**	**250.56 ± 160.35**	**0.02**

Glutamate	325.21 ± 160.97	305.92 ± 120.57	0.50

Methionine	21.92 ± 68.40	22.84 ± 47.48	0.93

**Histidine**	**167.69 ± 77.61**	**134.20 ± 46.86**	**0.02**

Arginine	117.38 ± 47.40	110.61 ± 52.52	0.50

**Tyrosine**	**81.49 ± 36.55**	**61.78 ± 24.87**	**0.002**

**Tryptophane**	**107.20 ± 36.72**	**86.90 ± 32.26**	**0.004**

			

**Sum**	**3283.91 **± **1137.04**	**2752.67 **± **692.84**	**0.0062**

Values (μM) are given in mean +/- standard deviation.

The correlation matrix (table 3) for the individual variables showed a strong correlation between the C_16 _and C_18 _sphingoid base metabolites. Also, doxSA and doxSO correlated well with each other but less so with the other sphingoid bases. For doxSO a weak but significant correlation with C_16 _and C_18_SA was seen, but not with the much more abundant C_16 _and C_18_SO. This indicates that DSB generation is metabolically independent from C_16 _and C_18_-sphingoid base formation. In relation to other markers we observed a correlation of the C_16 _and C_18 _sphingoid bases with total-as well as HDL-and LDL-cholesterol. DSBs, in contrast, showed a significant correlation with BMI and triglycerides (TG) and to a certain extent with LDL, but not with HDL.

**Table 3:** Correlation matrix of the analyzed variables.

	C_16_SO	C_16_SA	C_18_SO	C_18_SA	doxSO	doxSA	AGE	BMI	CHOL	HDL	LDL	TG	CRP
C_16_SO		0.43**	0.75**	0.36**	0.07	0.01	-0.12	-0.23*	0.22*	0.27**	0.18	-0.09	-0.10

C_16_SA	0.43**		0.57**	0.83**	0.27**	0.10	0.03	0.01	0.36**	0.20*	0.35**	0.13	-0.10

C_18_SO	0.75**	0.57**		0.65**	0.16	0.08	-0.06	-0.15	0.35**	0.36**	0.34**	-0.10	-0.12

C_18_SA	0.36**	0.83**	0.65**		0.34**	0.21	0.09	0.05	0.49**	0.31**	0.48**	0.14	-0.03

doxSO	0.07	0.27**	0.16	0.34**		0.76**	0.05	0.32**	0.14	0.00	0.20*	0.29**	0.11

doxSA	0.01	0.10	0.08	0.21	0.76**		0.00	0.33**	0.23*	0.03	0.25*	0.27**	0.18

AGE	-0.12	0.03	-0.06	0.09	0.05	0.00		0.12	-0.07	-0.05	-0.04	-0.06	0.12

BMI	-0.23*	0.01	-0.15	0.05	0.32**	0.33**	0.12		-0.27*	-0.45**	-0.11	0.04	0.41**

CHOL	0.22*	0.36**	0.35**	0.49**	0.14	0.23*	-0.07	-0.27*		0.49**	0.92**	0.44**	-0.03

HDL	0.27**	0.20*	0.36**	0.31**	0.00	0.03	-0.05	-0.45**	0.49**		0.23*	-0.14	-0.19

LDL	0.18	0.35**	0.34**	0.48**	0.20*	0.25*	-0.11	-0.11	0.92**	0.23*		0.33**	0.03

TG	-0.09	0.13	-0.10	0.14	0.29**	0.27**	0.04	0.04	0.44**	-0.14	0.33**		0.08

CRP	-0.10	-0.10	-0.12	-0.03	0.11	0.18	0.12	0.41**	-0.03	-0.19	0.03	0.08	

The pearson correlations were calculated for the whole dataset combining control and diabetic group. (Correlations ≥ 0.3 are highlighted bold, * p < 0.05, **p < 0.01)

To gain further insight into the physiology of the DSBs we analyzed their distribution into plasma lipoprotein fractions. Lipoproteins were separated by ultracentrifugation, fractionated and the individual fractions analyzed for cholesterol, triglycerides (TG) and sphingoid bases (figure 2D). The C_16_-and the C_18_-sphingolipids were found in the LDL and HDL but not in the VLDL fraction. In contrast, the DSBs were predominantly found in LDL and VLDL but were nearly absent in HDL. This indicates that the plasma DSBs are of hepatic origin since LDL and VLDL but not HDL primarily originate from the liver.

## Discussion

In this study we demonstrate that deoxy-sphingoid bases are elevated in patients with diabetes whereas the other analysed sphingoid base metabolites like C_16_SA, C_16_SO, C_18_SA and C_18_SO were not different. This indicates that impairments in glucose metabolism as it is the base of diabetes are reflected in changes of some but not all sphingoid bases. Sphingolipid metabolism represents a metabolic cross point which interconnects lipid (acyl-CoA) and amino acid (serine and alanine) metabolism. The cellular serine and alanine production is functionally linked to carbohydrate metabolism by their precursors 3-phosphoglycerate and pyruvate and thereby indirectly also to the carbohydrate metabolism. Therefore, the observed changes indicate a functional interaction between sphingolipid, glucose and fatty acid metabolism which results in an increased production of DSBs in diabetes.

A limitation of this study is the overrepresentation of patients receiving cholesterol lowering drugs in the diabetic group compared to the controls. Thus an impact of statin treatment on the sphingoid base levels cannot be completely excluded. However, diabetic patients with and without statin treatment show no significant differences in the sphingoid base levels. This indicates that a statin treatment does not significantly influence sphingoid base levels. Nevertheless, the influence of statins needs to be addressed in more detail in further studies.

Overall plasma amino acid levels were found to be lower in the diabetic group compared to controls. Among the most significantly lowered amino acids were threonine and serine. Plasma alanine levels, in contrast, were not significantly different between the two groups. It was recently shown that increasing L-alanine levels stimulate DSB formation, whereas the presence of L-serine suppresses DSB formation and increases the generation of C_16 _and C_18 _sphingoid bases [12]. The lower serine levels in the diabetic group might therefore explain why DSB levels were higher in the diabetics, since the alanine to serine ratio was decreased in these patients. However, lower levels of serine should then also be associated with lower C_18 _and C_18 _sphingoid base levels. This was, by trend, indeed the case although the differences between the two groups were not significant for this cohort.

Earlier reports on amino acid metabolism in obese patients indicate that the situation might be more complex, however. The observation that DSBs are preferentially found in LDL indicates that the plasma DSB levels are primarily of hepatic origin. It has been reported that alanine uptake into the liver is significantly increased in obese individuals [16]. Increased alanine uptake into the liver might thus result in increased DSB formation. However, due to the physiological alanine/glucose shuttle between muscle and liver, the hepatic uptake of alanine is not necessarily reflected in generally lower alanine levels in the venous blood [16].

Besides the direct uptake of alanine into the liver, another mechanism might also contribute to increased hepatic DSB generation. Hepatic glucose uptake is primarily mediated by GLUT2 and is hence insulin independent. Hyperglycemic conditions are therefore associated with high hepatic glucose levels, leading to increased glycolytic flux and production of pyruvate. Hepatic glucose overload might therefore lead to a build-up of pyruvate and its anaerobic conversion into either lactate or alternatively alanine which, in turn, is then converted to doxSA by the hepatic SPT.

This mechanism could also lead to increased DSB generation in other tissues in which glucose uptake is insulin independent, e.g. in kidney, pancreatic beta cells or neurons.

Especially in peripheral neurons elevated DSB levels could contribute to the pathology of diabetic sensory neuropathy (DSN). The inherited neuropathy HSAN1 is caused by a pathological overproduction of DSBs due to several missense mutations in SPT [4]. Interestingly, HSAN1 and DSN are clinically very similar: both have a late onset and slow progression and typically affect the distal extremities first. All peripheral nerves are affected, including pain fibers, motor neurons and autonomic nerves. The degeneration of small sensory fibers results in the loss of pain sensation, which in turn leads to painless injuries. Both HSAN1 and DSN are associated with skin ulcers, which is not a common feature in other peripheral neuropathies. The pathological background of DSN is not yet fully understood. Several theories have been discussed and might contribute to unravelling the pathology of DSN. This includes microangiopathy which results in neuronal ischemia, the formation of advanced glycated end products (RAGE) due to a non enzymatic glycosylation of proteins, chronic PKC activation or the increased generation of sorbitol via the polyol pathway, causing oxidative and osmotic stress [17,18]. Considering the neurotoxic properties of DSBs, it might therefore be conceivable that the elevated DSB levels in diabetic patients also contribute to the pathology of DSN.

However, in the context of our results, further studies are necessary to validate the role of atypical sphingoid bases as novel biomarkers in diabetes and to address a potential involvement of these metabolites in the pathology of DSN.

## Competing interests

The authors declare that they have no competing interests.

## Authors' contributions

MB performed the lipids analysis; MF performed the amino analysis; AO performed the statistical analysis; JMJ and MH were involved in study samples collection; AE was involved in the clinical chemistry analytics; TH participated an the design of the study and wrote the manuscript.

All authors have read and approved the final manuscript
